# A Novel Parallel-Preheating Supercritical CO_2_ Brayton Cycle for Waste Heat Recovery from Offshore Gas Turbines: Energy, Exergy, and Economic Analysis Under Variable Loads

**DOI:** 10.3390/e28010106

**Published:** 2026-01-16

**Authors:** Dianli Qu, Jia Yan, Xiang Xu, Zhan Liu

**Affiliations:** 1 School of Mechanics and Civil Engineering, China University of Mining and Technology, Xuzhou 221116, China; 2 Institute of Engineering Thermophysics, Chinese Academy of Sciences, Beijing 100191, China

**Keywords:** supercritical carbon dioxide, parallel-preheating Brayton cycle, gas turbine, waste heat recovery, 3E analysis, variable loads

## Abstract

Supercritical carbon dioxide (SC-CO_2_) power cycles offer a promising solution for offshore platforms’ gas turbine waste heat recovery due to their compact design and high thermal efficiency. This study proposes a novel parallel-preheating recuperated Brayton cycle (PBC) using SC-CO_2_ for waste heat recovery on offshore gas turbines. An integrated energy, exergy, and economic (3E) model was developed and showed good predictive accuracy (deviations < 3%). The comparative analysis indicates that the PBC significantly outperforms the simple recuperated Brayton cycle (SBC). Under 100% load conditions, the PBC achieves a net power output of 4.55 MW, while the SBC reaches 3.28 MW, representing a power output increase of approximately 27.9%. In terms of thermal efficiency, the PBC reaches 36.7%, compared to 21.5% for the SBC, marking an improvement of about 41.4%. Additionally, the electricity generation cost of the PBC is 0.391 CNY/kWh, whereas that of the SBC is 0.43 CNY/kWh, corresponding to a cost reduction of approximately 21.23%. Even at 30% gas turbine load, the PBC maintains high thermoelectric and exergy efficiencies of 30.54% and 35.43%, respectively, despite a 50.8% reduction in net power from full load. The results demonstrate that the integrated preheater effectively recovers residual flue gas heat, enhancing overall performance. To meet the spatial constraints of offshore platforms, we maintained a pinch-point temperature difference of approximately 20 K in both the preheater and heater by adjusting the flow split ratio. This approach ensures a compact system layout while balancing cycle thermal efficiency with economic viability. This study offers valuable insights into the PBC’s variable-load performance and provides theoretical guidance for its practical optimization in engineering applications.

## 1. Introduction

Gas turbines are extensively utilized across industries due to their compact footprint, rapid start-up capability, and lower emissions. However, the direct emission of high-grade exhaust gases from gas turbines generates significant environmental pollution while concurrently reflecting substantial inefficiencies in thermal energy utilization. To solve the issues of global environmental protection and the energy crisis [1], new requirements are imposed on power generation systems with environmentally friendly working media and high energy conversion efficiency. Compared with conventional coal-fired generation, the SC-CO_2_ Brayton cycle stands out in the next generation of power generation technology with unique advantages, such as better thermodynamic efficiency, radical system compactness, and exceptional operational flexibility. Moreover, the SC-CO_2_ Brayton cycle can also supply some additional deployment benefits in solar thermal and waste heat recovery contexts. At present, the SC-CO_2_ Brayton cycle technology has been successfully implemented across nuclear power generation [2,3,4], ship propulsion [5], waste heat utilization [6], and coal-fired power generation [7].

Until now, many researchers have conducted in-depth investigations on system configurations of the SC-CO_2_ Brayton cycle to pursue higher efficiency. The first SC-CO_2_ Brayton–Joule cycle was developed for nuclear reactor power generation [8]. In recent years, this technology has been proposed for applications in biomass [9], solar energy [10,11], and fossil fuel utilization [12]. Santini et al. [13] studied three types of Brayton cycle layouts using CO_2_ as a working fluid. The results showed that the thermal efficiency increased by 19.6% when transitioning from the regenerative cycle to the regenerative recompression cycle, and it further improved by 3.8% when evolving from the regenerative recompression cycle to the regenerative recompression reheating cycle. We will note that for the mid-temperature waste heat recovery application central to this study, adding a reheating stage, while offering marginal efficiency benefits, can significantly increase system complexity (requiring an additional reheater, potentially high-temperature valves, or a second turbine stage) and may lead to substantial increases in capital investment and maintenance costs. Among them, the net conversion efficiency of the regenerative recompression reheating CO_2_ cycle reached 34.04%. Manente et al. [14] conducted a performance comparison between a single-split double-expansion cycle and a double-split double-expansion cycle. The results showed that, compared with the traditional single regenerative cycle, the heat recovery efficiencies of the single-split and double-split double-expansion cycles increased by 3–7.7% and 17.8–28.5%, respectively. Darwin et al. [15] integrated a power generation unit of a simple cycle thermal power plant as a top cycle and combined it with a partially heated SC-CO_2_ Brayton cycle bottom cycle to construct an integrated thermal power plant system. Compared with traditional simple cycle thermal power plants, the total product unit cost of the system after integrating the SC-CO_2_ cycle was reduced by 37.38%, and the equipment was more compact and the operation and maintenance were much simpler. Maimoon et al. [16] researched an integration system of the SC-CO_2_ recompression Brayton cycle and a solar tower power station. They found that under higher temperature conditions, the integrated system demonstrated a higher thermodynamic efficiency, with the cycle thermal efficiency reaching 45.17%. Sun et al. [17] proposed two novel SC-CO_2_ cycle layouts (without additional bottoming cycles), and found that two novel cycles could achieve efficient recovery of waste heat from gas turbines by optimizing the arrangement of heat exchangers and turbines, and effectively reduce the additional costs brought by traditional combined cycle systems (such as those integrated with Organic Rankine Cycle (ORC)). Bahrami et al. [18] compared the supercritical Brayton cycle (SCBC), subcritical/trans-critical organic Rankine cycle (ORC), and Kalina cycle (KCS11) for waste heat recovery from micro gas turbines (MGTs) with an exhaust gas temperature of approximately 300 °C, and found that the trans-critical ORC (with R123 as the working fluid) exhibits the optimal comprehensive performance in terms of thermodynamic efficiency, net power improvement, and environmental impact, while the SCBC has the lowest levelized cost of electricity (LCOE) but inferior thermodynamic and environmental performance, and the KCS11 shows the worst overall performance. In addition, Bahrami et al. [18] conducted a comprehensive comparison of various bottoming cycles, including the supercritical CO_2_ cycle, along with an exploration of the interactions among different heat recovery strategies. Therefore, for specific application scenarios such as offshore platforms, the optimal heat allocation trade-off between bottoming-cycle power generation and topping-cycle regeneration constitutes a critical aspect of system design in determining overall energy utilization efficiency. Cao et al. [19] adopted a genetic algorithm to optimize a cascaded CO_2_ bottom cycle. The results showed that the efficiency of the cascaded cycle was 4.44% higher than that of the traditional steam Rankine cycle. Wang et al. [20] adopted a multi-objective optimization approach and analyzed the application scenarios of solar tower power plants. The results indicated that the intercooling cycle and the partial cooling cycle demonstrated the best system efficiency and specific work. Sharma et al. [21] integrated a SC-CO_2_ regenerative recompression Brayton cycle with marine gas turbines. It was shown that the overall system efficiency increased by 10%, and the net power could be increased up to 25% of the rated power, thereby verifying the feasibility and high efficiency of the regenerative recompression cycle in the application of marine waste heat recovery. Wang et al. [22] made performance comparisons among different trans-critical power cycles of CO_2_ and found that the two-stage trans-critical power cycle of CO_2_ had the highest net power output. Under the working condition of a 2928 kW engine with an exhaust temperature of 470 °C, the net power of the two-stage cycle reached 517.27 kW, which was 21.53% and 9.21% higher than that of the single-stage and three-stage cycles, respectively. Wright et al. [23] investigated the performance of three power cycles, namely the cascade cycle, the double regenerative cycle, and the preheating cycle, which were specifically designed for waste heat recovery. It was found that when compared to the simple recuperative Brayton cycle, the proposed three power cycles could generate an additional 1.2–1.6 MWe of electricity and increase power generation by approximately 22%. Kim et al. [24] clarified the applicability of different SC-CO_2_ cycles in waste heat recovery of small- and medium-sized LFG gas turbines. The results showed that the partial heating cycle became the preferred cycle form in recent times due to its simplicity and efficiency. After performance evaluation from the aspects of thermodynamic analysis, economic costs, and structural complexity, Li et al. [25] discovered that some partial heating cycles achieved an exergy efficiency of 55.08% with relatively simple structures. Alfani et al. [26] demonstrated that not all SC-CO_2_ cycle layouts are inherently suitable for waste heat recovery. Although the recompression recuperative cycle achieves a high degree of internal heat recuperation, its comparatively low heat source utilization efficiency ultimately results in suboptimal overall performance. In contrast, the simple recuperated cycle with a regenerator bypass exhibits a superior overall efficiency of approximately 27.5%.

As the waste heat temperature of gas turbines is highly compatible with SC-CO_2_ Brayton cycles, in-depth discussions have been conducted on the application of SC-CO_2_ Brayton cycle to gas turbine waste heat recovery. Bella et al. [27] conducted a thermodynamic analysis of an MT-30 gas turbine waste heat recovery SC-CO_2_ system. With the objective of maximizing the power output of the ship’s prime mover propulsion system, the optimal operating parameters were identified, leading to a 20% increase in system output. Cao et al. [28,29] combined an SC-CO_2_ Brayton cycle with a trans-critical CO_2_ cycle to form a cascade cycle. They found that the performance of the cascade cycle was superior to that of the traditional gas turbine–steam Rankine combined cycle, with a system efficiency increase of 4.44% and a net output power increase of 0.80 MW. Du et al. [30] found that when a two-stage compression cycle was utilized for power generation, the system efficiency increased by 1.73% compared with the traditional single compression cycle. Meanwhile, the total heat exchanger volume and mass were reduced by 0.37 m^3^ and 0.86 t, respectively. Song et al. [31,32] effectively mitigated the inhibitory effect of high preheating temperature on regeneration efficiency by improving a preheated SC-CO_2_ cycle and integrating a regeneration branch design, significantly enhancing the performance of a diesel engine waste heat recovery system, while also achieving high efficiency and a compact structure. Wu et al. [33] found that with flue gas temperatures ranging from 200 to 500 °C, the net output power of a multi-stage compression cycle increased by 3.9% to 26.3% compared to a single-stage cycle, and the optimal working pressure decreased by 13.2% to 31.0%. Yang et al. [34] adopted a superstructure method and made simultaneous optimizations of system structure and operation parameters. The results showed that with the heat source temperatures varying from 400 to 600 °C, the optimal system structure had a net output power that is 4.09–6.94% higher than that of the dual-split dual-expansion cycle. To achieve efficient recovery of waste heat from marine gas turbines, Zhang et al. [35] coupled a high-temperature sub-cycle and a low-temperature sub-cycle, and designed a novel cascaded cycle structure. The results showed that under design conditions, compared with the traditional CO_2_ cycle, the net output power of this new cascade cycle increased by 5.8% (4978 kW), and the exergy efficiency improved by 5.9% (37.8%). To recover waste heat from the exhaust gas of gas turbines, Walnum et al. [36] designed bottoming cycles with CO_2_, including single-stage and two-stage compression structures. They found that the net efficiency of the combined cycle could be increased to 48.9% and 50.0%, respectively, by using single-stage and two-stage CO_2_ bottoming cycles, which were 10.6% and 11.7% higher than that of the simple gas turbine cycle.

Based on the comprehensive literature review, it is evident that the SC-CO_2_ Brayton cycle is a promising technology for gas turbine waste heat recovery. The supercritical CO_2_ Brayton cycle shows promise for gas turbine waste heat recovery, yet current research exhibits two key limitations: a focus on either simple recuperative cycles or highly complex configurations unsuitable for mid-temperature waste heat, and a lack of comprehensive analysis under realistic variable-load conditions. To address these gaps, this study proposes a novel parallel-preheating recuperated Brayton cycle (PBC), whose originality lies in the innovative parallel integration of a preheater with the recuperator. This distinct configuration enables more flexible and efficient recovery of residual flue gas heat, significantly reducing exhaust temperature and enhancing overall energy utilization without unduly increasing system complexity. An energy, exergy, and economic (3E) calculation model is established to investigate the operation performance of the proposed novel PBC. The performance discrepancies between the PBC and the SBC are investigated in detail. The optimal operating conditions of the PBC under different loads are further screened, and the performance variation rules of the PBC during variable-load operation of gas turbines are summarized.

This study aims to explore the superior performance of the novel PBC in the waste heat recovery of gas turbines and summarize the variation laws of system performance under variable-load operation conditions of gas turbines. The detailed article structure is introduced below. Section 2 describes the system compositions and the operating principles of the SBC and the PBC. Section 3 constructs 3E mathematical models of the two cycles from the aspects of energy, exergy, and economic analysis. Section 4 conducts comprehensive performance comparisons between the PBC and the SBC, demonstrating the performance advantages of the PBC. Section 5 further analyzes the optimal operating conditions of the PBC under variable-load conditions of gas turbines and summarizes the performance variation rules under the optimal operating case. Section 6 summarizes the main conclusions of this study. In general, this study could provide a theoretical basis and technical support for design optimization and variable-load operation strategies of gas turbine waste heat recovery systems. While constructing a PBC system presents certain engineering challenges, the key components required are feasible under current technological conditions. The core value of this work lies in providing a solid theoretical foundation and clear operational guidelines for future practical applications. The optimal operating parameters identified in this study can serve as clear design targets; the performance maps under varying operating conditions provide a basis for developing part-load control strategies; and the economic analysis offers crucial insights for investment decisions. These outcomes significantly reduce the risks in the subsequent concept design and engineering development phases, laying a strong foundation for the construction of prototype systems.

## 2. System Description

**Figure 1:**
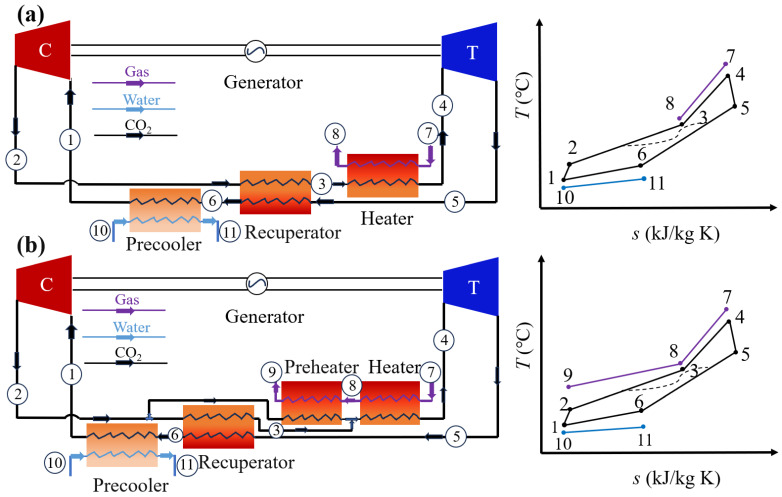
Cycle layout and corresponding *T*–s diagram: (**a**) SBC and (**b**) PBC.

To make full use of thermal energy in waste flue gas, a preheater is innovatively added to the SBC and arranged in parallel with the recuperator in this study. The proposed novel system is named the parallel-preheating recuperated Brayton cycle (PBC). With the addition of a preheater, the waste heat in the flue gas is more fully utilized by the PBC, and pinch temperature differences in the heater and the recuperator can be better controlled through the split ratio (α). In our simulations, the split ratio α was a key control variable dynamically adjusted to maintain the pinch-point temperature difference at approximately 20 K in both the recuperator and the preheater. The detailed system layouts and *T*–s diagrams of the SBC and the PBC are shown in Figure 1.

As Figure 1a shows, the operation of the SBC is introduced as follows. The low-temperature, low-pressure CO_2_ (1) is compressed into a high-temperature, high-pressure state (2) by the compressor and then enters the recuperator for preheating. The preheated CO_2_ (3) flows into the heater, where it absorbs waste heat from the gas turbine exhaust, further raising its temperature (4). Subsequently, the heated fluid expands through the turbine impeller to generate work. The exhaust fluid (5) returns to the recuperator, transferring its residual heat to the low-temperature fluid (6), before being cooled down by the precooler and completing the whole cycle. During this process, waste heat (7) from the gas turbine exhaust is transferred to the SC-CO_2_ power cycle via the heater. Figure 1b illustrates the system layout and T–s diagram of the PBC. Different from the SBC, once the working medium in the PBC is compressed by the compressor, it is divided into two parts according to the split ratio and introduced into the preheater and the regenerator, respectively, for heat absorption. As the T–s diagram of the PBC shows, the addition of the preheater enables more effective recovery of waste heat from the discharged flue gas, thereby reducing the exhaust gas temperature. After heat absorption, the two streams merge and subsequently enter the heater for further heating, raising the fluid temperature to a higher level (4). The heated CO_2_ then expands through the turbine, generating mechanical work while experiencing a pressure drop. The fluid exits the turbine at a lower pressure and temperature. The exhaust gas (5) flows through the recuperator, transferring its residual heat to the low-temperature fluid (6), which is then cooled by the precooler to complete the whole cycle. In the PBC, waste heat from the gas turbine flue gas is recovered via two heat exchangers, converting high-temperature flue gas (7) into low-pressure and low-temperature flue gas (9), thereby enhancing the overall utilization efficiency of flue gas waste heat. Compared to recompression cycles that require additional compressors, the PBC enhances performance merely by incorporating a preheating branch and a flow-split valve. This minimal architectural modification makes it a more feasible solution for retrofitting existing SBC systems.

## 3. Calculation Model

The Simulink software (2017b) is adopted to establish mathematical models of the PBC and the SBC from the aspects of energy, exergy, and economic analyses. The physical property data of flue gas from the gas turbine and CO_2_ are retrieved via the REFPROP 9.1 software. Within the parameter range studied, the differences in key property calculations between REFPROP 9.1 and version 10 are negligible and do not affect the robustness of the main conclusions regarding performance trends, relative improvements, or optimal operating points. The calculation model adopts a modular design, integrating component models into a single module. The modeled components include heat exchangers, compressors, turbines, and water pumps. To simplify the mathematical models of the two systems and achieve the intended analysis objectives, the following assumptions and constraints are made.

1. Kinetic energy, potential energy, heat losses, and frictional losses are ignored [37].
2. The neglect of kinetic and potential energy changes is standard practice as their contributions to the overall energy balance are negligible for the systems under consideration.
3. The SC-CO_2_ system operates in a steady state [38].
4. The pressure drop of the pipeline is ignored. The total pressure drop may reduce the system efficiency by 2% to 5%, but ignoring the pressure drop can significantly simplify the system configuration.
5. SC-CO_2_ undergoes adiabatic but non-isentropic processes in compressors and turbines [39]. Assuming constant isentropic efficiencies simplifies calculations and provides a valid, widely used approach for comparative analysis within the studied parameter ranges.
6. Cooling water under ambient conditions is utilized in the precooler.

In the present study, the turbine inlet pressure (TIP) varies within a range of 12–20 MPa. The settings of other input parameters are listed in Table 1.

### 3.1. Energy Model

The mass balance [43] equation is expressed as

(1) $$\underset{\mathrm{in}}{\sum }\dot{m}=\underset{\mathrm{out}}{\sum }\dot{m}$$

The energy balance equation is given as

(2) $$\dot{Q}-\dot{W}+\underset{\mathrm{in}}{\sum }\dot{m}h-\underset{\mathrm{out}}{\sum }\dot{m}h=0$$

The state at the compressor outlet is determined by the inlet parameters and the isentropic efficiency of the compressor.

(3) $$\eta_{\mathrm{cs}}=\frac{h_{2s}-h_{1}}{h_{2}-h_{1}}$$

where *η*_cs_ represents the isentropic efficiency of compressor, and *h*_i_ denotes the specific enthalpy of the working fluid at state point *i*.

The outlet state of the turbine is jointly determined by the inlet state of turbine, and its isentropic efficiency is expressed as

(4) $$\eta_{\mathrm{ts}}=\frac{h_{4}-h_{5}}{h_{4}-h_{5s}}$$

where *η*_ts_ represents the isentropic efficiency of the turbine.

For the PBC, the flow rate ($\dot{m}$_α_) through the recuperator depends on the system split ratio.

(5) $${\dot{m}}_{\alpha }={\dot{m}}_{2}\cdot \alpha$$

(6) $${\dot{m}}_{\beta }={\dot{m}}_{2}-{\dot{m}}_{\alpha }$$

where *α* represents the proportion of fluid flow entering the recuperator to the total flow, *m*_α_ represents the flow rate of the working medium passing through the recuperator, and $\dot{m}$_β_ represents the flow rate of working medium passing through the preheater.

The net electricity generation (*W*_net_) is defined as the difference between the total power generated by the turbine and the total power consumed by the compressor, and expressed as

(7) $${\dot{W}}_{\mathrm{net}}={\dot{W}}_{t}-{\dot{W}}_{\mathrm{com}}$$

where *W*_t_ refers to the output power of the SC-CO_2_ turbine, and *W*_com_ refers to the power consumption of the SC-CO_2_ compressor.

For the SBC, the thermal efficiency (*η*_th,s_) can be defined as

(8) $$\eta_{\mathrm{th},s}=\frac{{\dot{W}}_{\mathrm{net}}}{Q_{h}}$$

where *Q*_h_ refers to the input energy in the heater.

For the PBC, the thermal efficiency (*η*_th,p_) is defined as

(9) $$\eta_{\mathrm{th},p}=\frac{{\dot{W}}_{\mathrm{net}}}{Q_{h}+Q_{\mathrm{pre}}}$$

where *Q*_pre_ refers to the input energy in the preheater.

### 3.2. Exergy Model

The total exergy at the *k* point is given as

(10) $$\dot{E}x_{k}={\dot{m}}_{k}\left[\left(h_{k}-h_{0}\right)-T_{0}\left(s_{k}-s_{0}\right)\right]$$

The circulating exergy efficiency (*η*_ex_) is expressed as

(11) $$\eta_{\mathrm{ex}}=\frac{{\dot{W}}_{\mathrm{net}}}{{\dot{W}}_{t}+{\dot{E}}_{\mathrm{in}}}$$

where ${\dot{E}}_{in}$ refers to the total input exergy of the different cycles.

The exergy input is defined as

(12) $${\dot{E}}_{\mathrm{in}}={\dot{m}}_{{\mathrm{co}}_{2}}c_{p}\left(T_{\mathrm{in}}-T_{\mathrm{out}}-T_{0}\ln\frac{T_{\mathrm{in}}}{T_{\mathrm{out}}}\right)$$

where *c*_p_ refers to the constant-pressure specific heat capacity of the flue gas, and *T*_in_ and *T*_out_ denote the inlet and outlet temperatures of the flue gas, respectively. The specific values of the gas turbine’s flue-gas mass flow rate and exhaust temperature are provided in Table 1.

Table 2 shows the thermodynamic balance equations of each component in the two cycles.

### 3.3. Economic Model

The Module Costing Technique (MCT) proposed by Bailie et al. [44] is adopted to calculate the Equipment Investment Cost. MCT is commonly used to estimate the cost of a new plant, originally proposed by Guthrie [45] in the late 1960s and early 1970s of the 20th century, and modified by Ulrich [46]. Generally, MCT is considered to be the best way to estimate initial costs of plants. MCT correlates component cost with its basic material purchase cost, while also accounting for equipment material, type, operating pressure, and temperature.

The basic equipment cost is given as

(13) $$log_{10}C_{p}^{0}=K_{1}+K_{2}log_{10}X+K_{3}{\left(log_{10}X\right)}^{2}$$

where *K*_1_, *K*_2_, and *K*_3_ denote cost-fitting coefficients. Specifically, for the heat exchangers, *X* represents the heat transfer area with a unit of m^2^, whereas for compressors, turbines, and water pumps, *X* refers to power rating in kilowatts (kW).

Accounting for the influence of construction materials and operating pressures across different equipment, the capital cost is modified as

(14) $$C_{\mathrm{bm}}=C_{p}^{0}(B_{1}+B_{2}F_{p}F_{m})$$

where *F*_m_ and *F*_p_ are the cost-correction factors for material and pressure, respectively.

*F*_p_ is calculated by the following formula:

(15) $$log_{10}F_{p}=C_{1}+C_{2}log_{10}p+C_{3}{\left(log_{10}p\right)}^{2}$$

where *p* is the design pressure (MPa) on the high-pressure side of the corresponding component, with a unit of bar, and *C*_1_, *C*_2_, and *C*_3_ are empirical fitting coefficients.

The fitting coefficients are presented in Table 3.

In order to estimate the area of the heat exchangers, the approximate values of the overall heat transfer coefficient are considered. For the heater and the preheater, a compact finned-tube heat exchanger is adopted, with a heat transfer coefficient set to 1.6 kW/(m^2^⋅K); the regenerator uses a finned heat exchanger with a sinusoidal channel core, and its heat transfer coefficient is 3.0 kW/(m^2^⋅K); the precooler employs a combination of a printed circuit heat exchanger (PCHE) and a seawater plate heat exchanger, with the heat transfer coefficient specified as 2.0 kW/(m^2^⋅K) [48,49].

The heat exchange area of the different heat exchange sections is given as

(16) $$A_{i}=\frac{Q_{i}}{U_{i}\Delta T_{i}}$$

where *U*_i_ refers to the overall heat transfer coefficient of the heat exchanger.

The logarithmic mean temperature difference [50] is adopted here.

(17) $$\Delta T_{i}=\frac{\Delta T_{i,\max}-\Delta T_{i,\min}}{\ln\frac{\Delta T_{i,\max}}{\Delta T_{i,\min}}}$$

Since the reference data of the cost figures is taken from 2001, the impact of time-influencing factors, such as inflation, on costs should be considered. Hence, the Chemical Engineering Plant Cost Index (CEPCI) is introduced for revision, with *CEPCI*_2001_ = 397 and *CEPCI*_2025_ = 804. As SC-CO_2_ is treated as the working fluid, the corresponding coefficients in the equipment cost calculations are selected based on Danieli’s work [40,51].

(18) $$c_{\mathrm{reference}}=c_{\mathrm{original}}\frac{CEPCI_{\mathrm{reference}}}{CEPCI_{\mathrm{original}}}$$

The total capital cost of the equipment is

(19) $$C_{\mathrm{tot}}=\frac{CEPCL_{2025}}{CEPCL_{2001}}\left(C_{\mathrm{bm},h}+C_{\mathrm{bm},\mathrm{pre}}+C_{\mathrm{bm},\mathrm{re}}+C_{\mathrm{bm},t}+C_{\mathrm{bm},\mathrm{com}}+C_{\mathrm{bm},\mathrm{pre}}+C_{\mathrm{bm},\mathrm{cool}}\right)$$

where *C*_bm,h_, *C*_bm,pre_, *C*_bm,re_, *C*_bm,t_, *C*_bm,com_, *C*_bm,pre_, and *C*_bm,cool_ denote the capital costs of the heater, the preheater, the recuperator, the turbine, the compressor, the water pump, and the precooler, respectively.

The capital recovery factor is expressed as [51,52]

(20) $$CRF=\frac{i{\left(1+i\right)}^{LT}}{\left[{\left(1+i\right)}^{LT}-1\right]}$$

where *i* refers to the depreciation rate, set to 5%, and *LT* is the life cycle of the power plant, with a value of 20 years [53,54].

The cost of electricity production is determined by

(21) $$\mathrm{EPC}=\frac{C_{\mathrm{tot}}CRF+COM}{t_{\mathrm{op}}W_{\mathrm{net}}}$$

where *COM* is the operation and maintenance cost, set to 1.5%, and *t*_op_ refers to the run time, with a value of 8000 h.

The dynamic payback period is expressed as

(22) $$\mathrm{PDD}=\ln\left(\frac{t_{\mathrm{op}}W_{\mathrm{net}}C_{\mathrm{elec}}-COM}{t_{\mathrm{op}}W_{\mathrm{net}}C_{\mathrm{elec}}-COM-i\cdot C_{\mathrm{tot}}}\right)/\ln(1+i)$$

where *C*_elec_ represents the grid-connected tariff, with a value of 0.6 CNY/kWh [55].

The flue-gas characteristics of the AGT-12 gas turbine under varying load conditions are summarized in Table 4, and the operational data utilized in this study for the gas turbine were procured from industrial partners.

### 3.4. Model Validation

**Figure 2:**
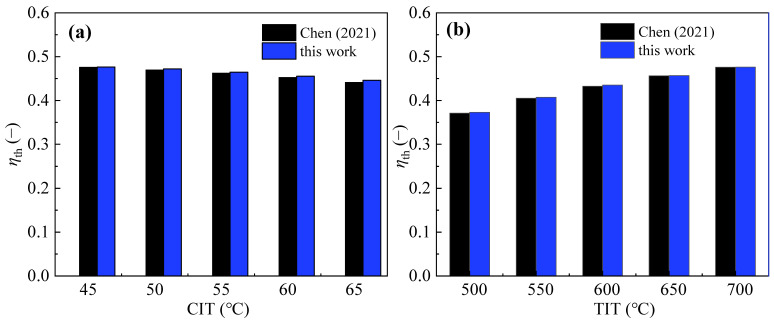
Comparison of the thermal efficiency of the SBC: (**a**) CIT and (**b**) TIT [56].

The test results of the SBC are adopted to validate the calculation model introduced above. The initial settings of the SBC in ref. [56] are listed in Table 5. With the same settings, the thermal efficiency of the SBC under different CITs and TITs is calculated by the present mathematic model, and the detailed result comparisons are shown in Figure 2. It is evident that under the same input parameters, the prediction deviations between the present model and the reference model are less than 3%. The PBC is based on the validated SBC model, with the addition of a preheater branch, keeping the core component models unchanged. This inheritance from the SBC model ensures that the PBC framework can be reliably used for further calculations. Additionally, the performance improvement trends of the PBC, such as increased net power and efficiency, align qualitatively with advancements in SC-CO_2_ cycles with added heat transfer stages, reinforcing the physical rationale behind the PBC’s gains. Thus, while direct experimental validation is not available, the PBC’s credibility is supported by the successful SBC model validation and qualitative consistency with the literature, providing a solid foundation for future calculations. Hence, the present calculation is acceptable and reliable and can be used for the following performance prediction of the two dynamic cycles.

## 4. Performance Comparison Between the SBC and the PBC

### 4.1. Thermodynamic Analysis

With turbine inlet pressure (TIP) ranging from 12 MPa to 20 MPa and compressor inlet pressure (CIP) ranging from 8.2 MPa to 9.0 MPa, comparisons of thermodynamic performance and *η*_ex_ between the SBC and the PBC were investigated.

**Figure 3:**
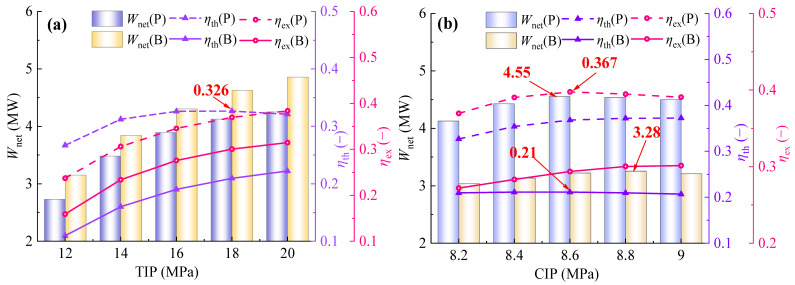
Comparison of *W*_net,_ *η*_th_ and *η*_ex_ between SBC and PBC: (**a**) TIP and (**b**) CIP.

As shown in Figure 3a, *W*_net_ and *η*_ex_ of both SBC and PBC gradually increase with the rise in TIP. According to Equations (7)–(9), when TIP increases, the power generation rates of the turbine in both systems grow much faster than the power consumption rate of the compressor, thus *W*_net_ of the two systems shows an upward trend, indicating that increasing TIP is beneficial for enhancing the power generation capacity of the SBC and the PBC. Meanwhile, *η*_ex_ of both systems also continuously increases with the rise in TIP, suggesting that the elevation of TIP effectively enhances the utilization of available energy in flue gas, thereby improving the energy utilization efficiency of power cycles. As for *η*_th_, it exhibits a monotonically increasing trend in the SBC, while it first increases and then decreases in the PBC, with a distinct maximum value of 0.326. This provides a theoretical basis for choosing the optimal TIP. Compared with the SBC, the average values of *W*_net_, *η*_th_, and *η*_ex_ of the PBC are increased by about 20.3%, 37.5%, and 22.5%, respectively. This level of performance enhancement is significant when compared to other cycle modifications. For example, while recompression cycles can achieve higher gains [13], the PBC accomplishes this with a much simpler system architecture, akin to the philosophy of partial heating cycles [23,24], but with a more pronounced performance boost. Thus, within the variation range of TIP, the thermodynamic performance of the PBC significantly outperforms that of SBC.

As shown in Figure 3b, *W*_net_ of both systems initially increases and then decreases with the rise in CIP. *W*_net_ of the PBC reaches its maximum value at 8.6 MPa, while that of the SBC peaks at 8.8 MPa. Within this range, *W*_net_ of the PBC is consistently significantly higher than that of SBC, indicating that the PBC has a more advantageous power generation capacity within the CIP variation range. In terms of *η*_th_, it shows a monotonically increasing trend with the increase in CIP in the PBC, while it first increases and then decreases in the SBC, reaching a minimum value at 8.6 MPa. The gap between the two systems gradually widens thereafter. With the increase in CIP, *η*_ex_ also experiences a variation of first increase and then decrease in PBC, reaching its peak at 8.6 MPa. In the SBC, *η*_ex_ continues to rise. The gap between the two cycles gradually narrows after 8.6 MPa, but *η*_ex_ of the PBC is always higher than that of the SBC. From the overall thermodynamic performance, *W*_net_, *η*_th_, and *η*_ex_ of the PBC are increased by 27.5%, 45.9%, and 25.6% on average compared to those of the SBC.

### 4.2. Economic Analysis

The economic performance comparison between PBC and SBC systems was investigated across the ranges of TIP and CIP.

**Figure 4:**
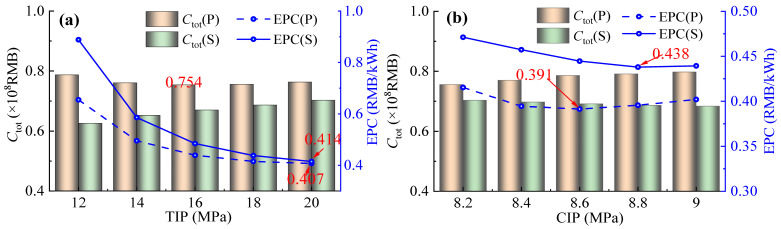
Comparison of *C*_tot_ and EPC between SBC and PBC: (**a**) TIP and (**b**) CIP.

As shown in Figure 4a, with the increase in TIP, the total initial investment (*C*_tot_) of SBC progressively rises, while that of PBC initially decreases and then increases. Based on Equation (19), *C*_tot_ represents the sum of component investment costs. Specifically, costs of the compressor, turbine, and heater increase monotonically. This is mainly because, for rotating equipment, *C*_bm,com_ and *C*_bm,t_, which are determined primarily by power ratings (MCT), escalate with power requirements. Moreover, for heaters, larger heat transfer areas are required for increased heat transfer load combined with a reduced logarithmic mean temperature difference, which increases the value of *C*_bm,heat_. Conversely, costs of the preheater, precooler, and recuperator decrease with rising CIP due to a reduced heat exchange amount and a smaller heat exchange area. The monotonic increase of *C*_tot_ in the SBC is mainly attributed to the fact that the combined cost escalations from compressors, turbines, and heaters outweigh the reductions from precoolers and recuperators. As for the PBC, when *C*_tot_ decreases below 18 MPa, the aggregate cost reductions from the preheater, precooler, and recuperator, exceed the increases from the compressor, turbine, and heater. When the subsequent *C*_tot_ increases above 18 MPa, *C*_bm,pre_ becomes dominant. In addition, variations in electricity production cost (EPC) values of the PBC and the SBC are investigated. It can be seen that both decrease monotonically with an increase in TIP. As the EPC of the SBC shows a faster decline rate, the EPC gap between the PBC and the SBC gradually narrows. However, when TIP varies from 12 MPa to 20 MPa, the EPC of the PBC is consistently found to be lower than that of the SBC, achieving an average reduction of 12%.

As illustrated in Figure 4b, with the increase in CIP, *C*_tot_ of the SBC increases monotonically, while that of the PBC decreases monotonically. This phenomenon can be attributed to a gradual increase in the system mass flow rate of CO_2_ (*m*_co2_) with rising CIP, which leads to higher power requirements for both compressors and turbines, thereby increasing the values of *C*_bm,com_ and *C*_bm,t_. For the heater, *C*_bm,heat_ increases because the growth rate of heat transfer load exceeds the decline rate of the average temperature difference. In contrast, other heat exchangers show an opposite change trend. Since the cumulative increase in the former three components exceeds the reduction in the latter three, *C*_tot_ of the PBC gradually rises. As for the SBC, both *C*_bm,com_ and *C*_bm,t_ decreases with the increase in CIP, while the investment cost of heat exchangers increases. However, the cost increment in the heat exchanger is smaller than the cost reductions in the compressor and turbine, resulting in a monotonic decrease in *C*_tot_ of the SBC, though the decline margin is minimal. In terms of EPC, the PBC consistently maintains a lower value compared to SBC, primarily due to its significantly higher *W*_net_. The EPC of the PBC first decreases and then increases, reaching a minimum at CIP = 8.6 MPa. The gap between the two systems gradually narrows when CIP exceeds 8.6 MPa. Within CIP ranging from 8.2 MPa to 9.0 MPa, the PBC achieves an average EPC reduction of 19.1% compared to the SBC. This economic improvement underscores the viability of the PBC.

**Figure 5:**
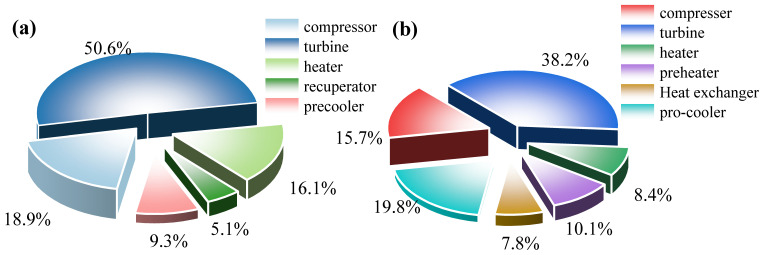
Investment proportion of each component: (**a**) SBC and (**b**) PBC.

Figure 5 shows the investment proportion of each component in the SBC and the PBC, with TIP = 18 MPa and CIP = 8.6 MPa. It is easy to see that the turbine accounts for the highest cost proportion in both the SBC (50.6%) and the PBC (38.2%). The compressor accounts for the second-highest cost proportion, 18.9% in the SBC. However, with the addition of a preheater in the PBC, the cost proportion of the compressor decreases to 15.7%, and the precooler achieves the second-highest cost proportion, 19.8%.

While the PBC offers superior thermodynamic and economic performance, it presents certain drawbacks compared to the SBC, primarily due to the added preheater and associated components. This increases system complexity and initial investment, leading to an average rise of approximately 17.16% in total capital cost. However, in space-constrained environments such as offshore platforms, the system’s footprint concern is now commonly mitigated by increasing the preheater’s hot-side outlet temperature and reducing the temperature approach. This approach effectively minimizes the heat exchanger’s size, enhancing the PBC’s suitability for applications where space is limited.

### 4.3. Comprehensive Performance Comparison

**Figure 6:**
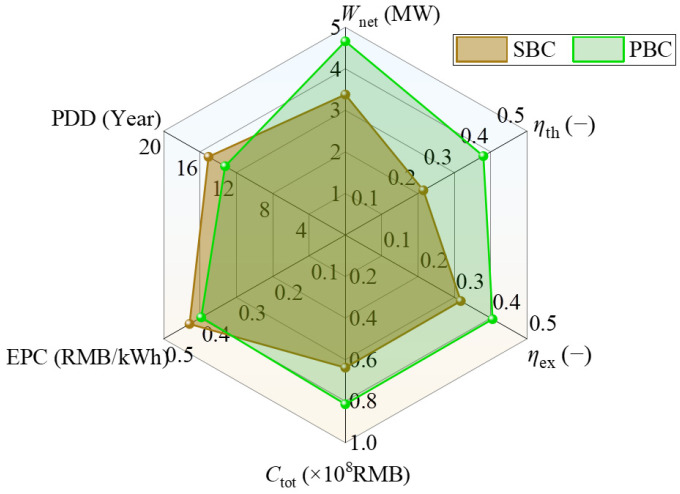
Comprehensive performance comparison of the SBC and the PBC.

Figure 6 presents comprehensive performance comparisons of SBCs and PBCs under TIP = 18 MPa and CIP = 8.6 MPa. It is clearly observed that under the same operating conditions, the reference coefficients such as *C*_tot_, *W*_net_, *η*_th_, and *η*_ex_ of the PBC are larger than those of the SBC, while parameters like dynamic payback period (PDD) and EPC of the PBC have smaller values compared to those of the SBC. Larger *C*_tot_ is also due to the fact that the PBC has an additional preheater. In terms of the meanings of PDD and EPC, smaller PDD and EPC values imply higher economic benefits. Therefore, the PBC is encouraged to be adopted in actual engineering, and in-depth research on the PBC is necessary and significant.

## 5. Performance Analysis of the PBC Under Variable Loads of the Gas Turbine

Based on the above comparative analysis, it can be obtained that the PBC demonstrates more significant performance advantages in thermodynamic and economic aspects. Due to excellent thermal physical properties of SCO_2_, it shows high applicability in waste heat recovery and utilization in actual gas turbines. According to flue gas temperature and flow data of an AGT-12 gas turbine under different loads provided in Table 4, the comprehensive performance of the PBC under variable-load operations of the gas turbine is studied, and the design condition is optimized as well.

### 5.1. 100% Operation Load

**Figure 7:**
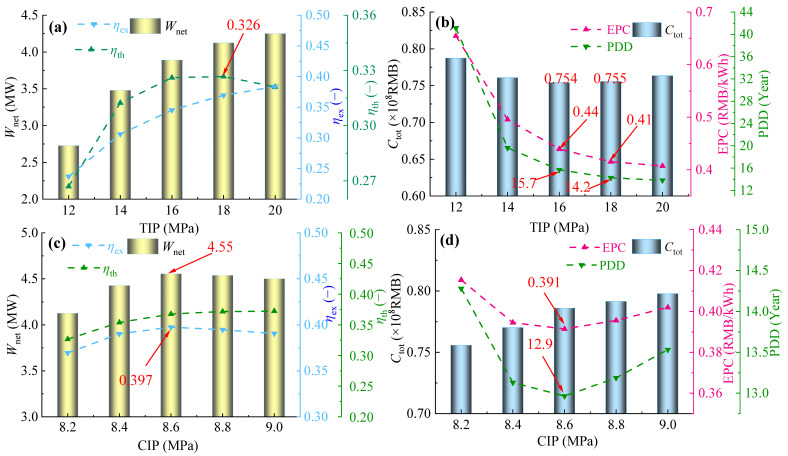
Variations in thermodynamic and economic parameters at 100% operation load: (**a**,**b**) TIP and (**c**,**d**) CIP.

Figure 7a,b presents variations in thermodynamic parameters (*W*_net_, *η*_th_, and *η*_ex_) and economic parameters (*C*_tot_, EPC, and PDD) of the PBC with TIP at 100% gas turbine load. It is easy to see that, with TIP ranging from 12 to 20 MPa, both *η*_ex_ and *W*_net_ of the PBC increase monotonically with rising TIP, while *η*_th_ first increases and then decreases, reaching its maximum at 18 MPa. Although there is minimal difference in *η*_th_ at TIP = 16 MPa and 18 MPa, further consideration is required for the selection of the optimal TIP−The “optimal” operating condition we reported refers to the set of parameters, among all the discrete combinations tested across a comprehensive and reasonable operating envelope, that yielded the best comprehensive performance (maximum net power output or minimum levelized cost of electricity). As for economic parameters, both PDD and EPC of PBC decrease monotonically with an increase in TIP, whereas *C*_tot_ first decreases and then increases, reaching a minimum of 0.754 at 16 MPa. *C*_tot_ comprises the sum of component investment costs. Costs of the compressor, turbine, and heater all increase monotonically. This is because coefficients *C*_bm,com_ and *C*_bm,t_ are primarily determined by their power ratings under MCT, and increasing system power will raise these costs. For heaters, heightened heat load coupled with reduced logarithmic temperature difference usually causes the requirement of a larger heat exchange area, which correspondingly increases *C*_bm,heat_. Conversely, preheaters, precoolers, and recuperators exhibit cost reductions due to decreased heat transfer area resulting from lower heat load at elevated CIP. EPC decreases monotonically because the growth rate of *W*_net_ consistently exceeds that of *C*_tot_. When TIP increases from 16 MPa to 18 MPa, *C*_tot_ increases by only 0.2%, while EPC and PDD decrease by 5.4% and 7.0%, respectively. Therefore, considering both thermodynamic and economic performance, the inlet pressure of 18 MPa is greatly recommended and identified as the optimal TIP under 100% gas turbine load.

With TIP = 18 MPa, the effect of CIP on thermodynamic and economic parameters of the PBC at 100% gas turbine load was investigated. As Figure 7c shows, when CIP increases from 8.2 MPa to 9.0 MPa, *η*_ex_ increases monotonically, while both *W*_net_ and *η*_th_ first increase and then decrease, with a maximum value achieved at CIP = 8.6 MPa. Therefore, from the perspective of thermodynamic performance, the optimal CIP is tentatively set at 8.6 MPa. Figure 7d shows variations in economic parameters with CIP. With the increase in CIP, *C*_tot_ shows a gradual increasing trend, while both EPC and PDD first decrease and then increase. This is because, when CIP < 8.6 MPa, the rate of increase of *W*_net_ is greater than that of *C*_tot_, and the opposite is true when CIP > 8.6 MPa. Therefore, from the perspective of economic performance, it is recommended to use 8.6 MPa as the optimal CIP. Combining the comparison of thermodynamic performance, 8.6 MPa is treated as the optimal CIP under 100% gas turbine load.

**Figure 8:**
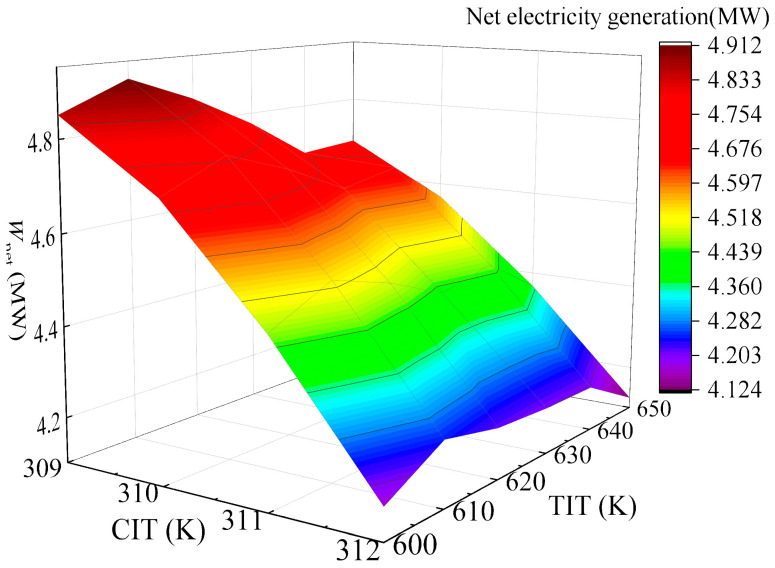
Variation of *W*_net_ with combined effects of CIT and TIT in 100% operation load.

The effects of compressor inlet temperature (CIT) and turbine inlet temperature (TIT) on system *W*_net_ were investigated under TIP = 18 MPa and CIP = 8.6 MPa. As Figure 8 shows, *W*_net_ undergoes a gradual decrease with the increase in CIT, while it initially increases and then decreases with rising TIT, reaching an extreme value at TIT = 610 K. This is mainly because an excessively small pinch temperature difference increases heat exchanger area and capital costs, whereas an excessively large pinch temperature difference induces significant exergy loss. To balance energy efficiency and economic feasibility, a pinch temperature difference of approximately 20 K is targeted. After calculation analysis, it is confirmed that at CIT = 310 K and TIT = 600 K, satisfactory power generation capacity is maintained while achieving the required 20 K pinch temperature difference. Consequently, CIT = 310 K and TIT = 600 K are selected as the optimal operating temperatures for the gas turbine under full-load conditions. At the optimal operation point, PBC delivers *W*_net_ of 4.72 MW, with *η*_th_ of 33.87% and *η*_ex_ of 40.9%. *C*_tot_ reaches 7.9 × 10^8^ CNY, yielding EPC of 0.38 CNY/kWh and a PDD of 12.52 years.

### 5.2. 75% Operation Load

**Figure 9:**
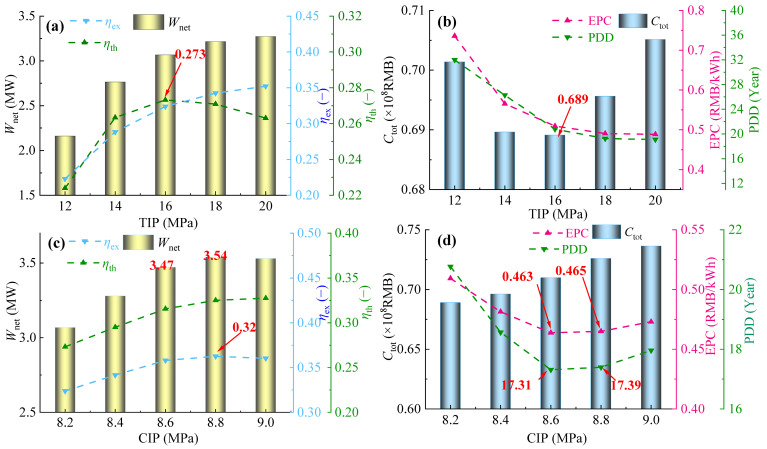
Variations in thermodynamic and economic parameters in 75% operation load: (**a**,**b**) TIP and (**c**,**d**) CIP.

Figure 9a,b presents variations in thermodynamic parameters (*W*_net_, *η*_th_, and *η*_ex_) and economic parameters (*C*_tot_, EPC, and PDD) of the PBC with changing TIP at 75% gas turbine load.

As Figure 9a shows, when TIP increases from 12 to 20 MPa, both *η*_ex_ and *W*_net_ of the PBC increase monotonically, while *η*_th_ first increases and then decreases, reaching its maximum value of 0.273 at TIP = 16 MPa. Both PDD and EPC decrease monotonically with the increase in TIP, whereas *C*_tot_ first decreases and then increases, reaching a minimum value of 0.689 at TIP = 16 MPa, as shown in Figure 9b. As for the monotonic decrease of EPC, it is mainly because the growth rate of *W*_net_ consistently exceeds that of *C*_tot_. Therefore, considering both thermodynamic and economic performance, 16 MPa is identified as the optimal TIP under 75% gas turbine load.

With an optimal TIP of 16 MPa, the influence of CIP on the thermodynamic and economic performance of the PBC under 75% load operation is studied. As Figure 9c shows, *η*_ex_ increases monotonically with an increase in CIP. Both *W*_net_ and *η*_th_ first increase and then decrease, with a maximum value achieved at CIP = 8.8 MPa. Therefore, from the perspective of thermodynamic performance, the optimal CIP is tentatively set at 8.8 MPa.

Figure 9d shows variations in economic parameters with CIP. As CIP increases from 8.2 to 9.0 MPa, *C*_tot_ gradually increases, and both EPC and PDD first decrease and then increase. This phenomenon arises because, when CIP < 8.8 MPa, the rate of increase of *W*_net_ is greater than that of *C*_tot_, and the opposite is true when CIP > 8.8 MPa. Therefore, from the perspective of economic performance, it is recommended to adopt 8.8 MPa as the optimal CIP. Combining the comparison of thermodynamic performance, the pressure 8.8 MPa is chosen as the optimal CIP under 75% gas turbine load.

**Figure 10:**
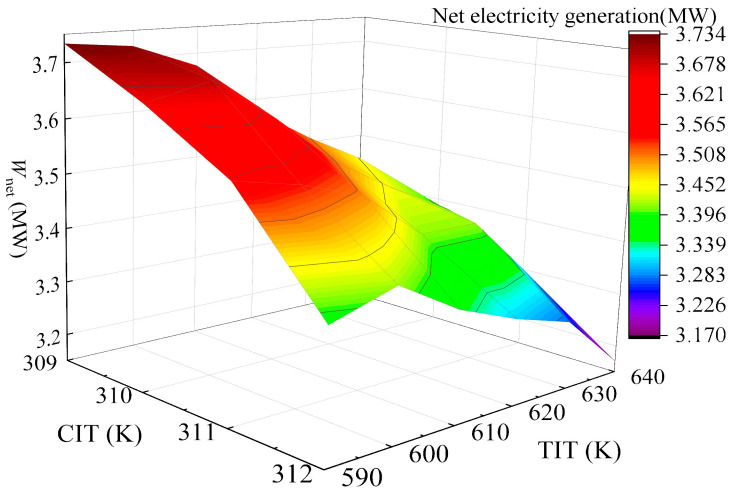
Variation of *W*_net_ with combined effects of CIT and TIT in 75% operation load.

Under TIP = 16 MPa and CIP = 8.8 MPa, the variation in system *W*_net_ with CIT and TIT was investigated and is shown in Figure 10. It is easy to see that *W*_net_ undergoes a gradual decrease with increasing CIT, while it initially increases and then decreases with rising TIT, reaching an extreme value at TIT = 600 K. Moreover, it is found that at CIT = 310 K and TIT = 600 K, satisfactory power generation capacity is maintained while achieving the required 20 K pinch temperature difference. Therefore, under the 75% load condition, the optimal operation temperatures of the gas turbine are determined to be CIT = 310 K and TIT = 600 K. At this operation point, the PBC delivers *W*_net_ of 3.68 MW, with *η*_th_ of 33.03% and *η*_ex_ of 37.51%, and *C*_tot_ reaches 7.72 × 10^8^ CNY, yielding an EPC of 0.47 CNY/kWh and a PDD of 18.12 years.

### 5.3. 50% Operation Load

**Figure 11:**
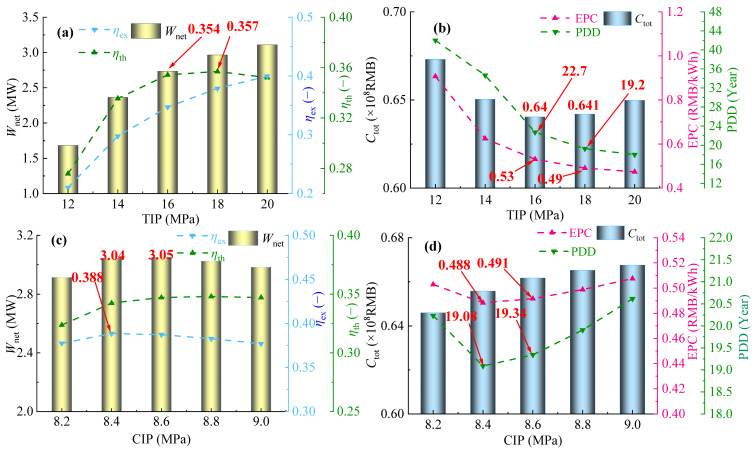
Variations in thermodynamic and economic parameters in 50% operation load: (**a**,**b**) TIP and (**c**,**d**) CIP.

While the gas turbine operation load is set at 50%, the related parameter variations in the PBC are investigated and shown in Figure 11a,b. It can be seen that within the TIP range of 12 to 20 MPa, both *η*_ex_ and *W*_net_ of the PBC increase monotonically, while *η*_th_ first increases and then decreases, reaching its maximum at 18 MPa. Meanwhile, both PDD and EPC of the PBC decrease monotonically with the increase in TIP, whereas *C*_tot_ first decreases and then increases, reaching a minimum at 18 MPa. When TIP increases from 16 to 18 MPa, *C*_tot_ increases by only 0.21%, while EPC and PDD decrease by 7.5% and 15.1%, respectively. Therefore, TIP = 18 MPa is economically recommended. Considering both thermodynamic and economic performance, 18 MPa is identified as the optimal TIP under 50% gas turbine load.

Figure 11c,d correspondingly displays variations in thermodynamic and economic parameters of the PBC with variable CIP under TIP = 18 MPa. As Figure 11c shows, With the increase in CIP, *W*_net_ shows a trend of first rising and then falling, reaching a maximum value of 3.05 MW at 8.6 MPa, which is approximately 0.3% higher than the power generation at 8.4 MPa. Meanwhile, *η*_ex_ also shows a similar trend, and reaches a peak of 38.8% at 8.4 MPa. Different from *W*_net_ and *η*_ex_, *η*_th_ increases monotonically with CIP. It is difficult to directly determine the optimal CIP from the thermodynamic performance alone. As Figure 11d shows, both EPC and PDD first decrease and then increase with the increase in CIP, reaching the minimum value at 8.4 MPa. When CIP increases from 8.4 MPa to 8.6 MPa, EPC and PDD increase by 0.6% and 1.4%, respectively. Therefore, considering the system performance comprehensively, 8.4 MPa is selected as the optimal CIP for the gas turbine at 50% operation load.

**Figure 12:**
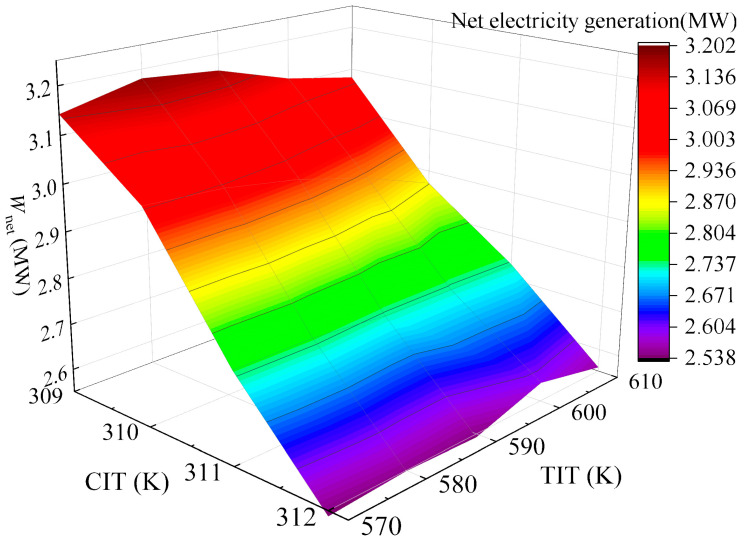
Variation of *W*_net_ with combined effects of CIT and TIT in 50% operation load.

The variation of *W*_net_ under TIP = 18 MPa and CIP = 8.4 MPa is presented as a function of both CIT and TIT and shown in Figure 12. It can be found that *W*_net_ experiences a gradual decrease with increasing CIT, while it initially increases and then decreases with rising TIT, reaching an extreme value at TIT = 580 K. To balance energy efficiency and economic feasibility, a pinch temperature difference of approximately 20 K is targeted. Satisfactory power generation capacity is maintained at CIT = 309 K and TIT = 580 K, while achieving the required 20 K pinch temperature difference. Therefore, the optimal operating temperatures for the gas turbine are focused at CIT = 309 K and TIT = 580 K under 50% operation load of the gas turbine. At this operation point, the system delivers *W*_net_ of 3.17 MW, with *η*_th_ of 30.97% and *η*_ex_ of 36.43%, and *C*_tot_ reaches 7.035 × 10^8^ CNY, yielding an EPC of 0.50 CNY/kWh and a PDD of 20.26 years.

### 5.4. 30% Operation Load

**Figure 13:**
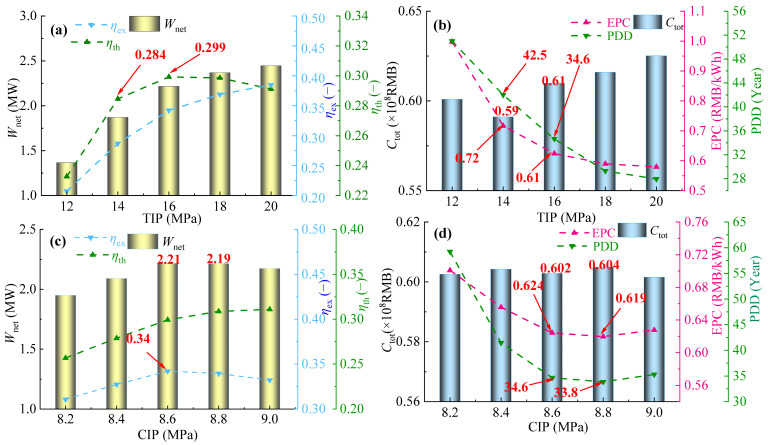
Variations in thermodynamic and economic parameters in 30% operation load: (**a**,**b**) TIP and (**c**,**d**) CIP.

When the gas turbine operates under a 30% load, the effect of TIP on the parameter variations in the PBC was investigated, and the detailed results are shown in Figure 13a,b. It can be seen that with the increase in TIP, *W*_net_ and *η*_ex_ show a monotonically increasing trend, while *η*_th_ first rises and then decreases, reaching a maximum value of 29.9% at 16 MPa. *W*_net_ increases by 5.01%, with TIP varying from 14 MPa to 16 MPa. As for EPC and PDD, both continuously decrease with the increase in TIP. *C*_tot_ first decreases and then increases, reaching the minimum value at 14 MPa. Compared with 14 MPa, *C*_tot_ at 16 MPa increases by 3.27%, while EPC and PDD decrease by 15.2% and 18.5%, respectively. Based on the comprehensive analysis of various performance indicators, it is reasonable to choose 16 MPa as the optimal TIP.

Figure 13c,d demonstrates variations in both thermodynamic and economic parameters with changing CIP under TIP = 16 MPa. As shown in Figure 13c, with the increase in CIP, both *W*_net_ and *η*_ex_ first increase and then decrease, reaching their maximum values of 2.21 MW and 34% at CIP = 8.6 MPa. Different from *W*_net_ and *η*_ex_, *η*_th_ increases monotonically with the increase in CIP. As can be seen from Figure 13d, both EPC and PDD continuously decrease with the increase in CIP, and *C*_tot_ reaches its minimum value at 8.6 MPa. When CIP ranges from 8.6 MPa to 8.8 MPa, *C*_tot_ increases by approximately 0.3%, while EPC and PDD decrease by 0.8% and 2.3%, respectively. Based on comprehensive thermodynamic performance analysis, it is reasonable to select 8.6 MPa as the optimal CIP for the PBC under the 30% load condition of the gas turbine.

**Figure 14:**
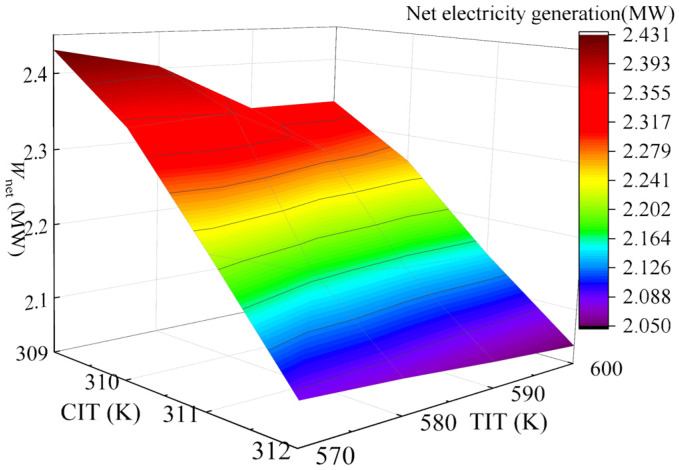
Variation of *W*_net_ with combined effects of CIT and TIT in 30% operation load.

The variation in system *W*_net_ under TIP = 16 MPa and CIP = 8.6 MPa with both CIT and TIT is depicted in Figure 14. Obviously, a gradual decrease in *W*_net_ with increasing CIT is observed. However, *W*_net_ initially increases and then decreases with rising TIT, reaching an extreme value at TIT = 580 K. To achieve a pinch temperature difference of approximately 20 K, detailed calculations and analysis were performed. It was confirmed that at CIT = 309 K and TIT = 580 K, satisfactory power generation capacity is maintained. Consequently, CIT = 310 K and TIT = 580 K are selected as the optimal operating temperatures for the gas turbine under the 30% load condition. At this operating point, the PBC delivers *W*_net_ of 2.32 MW, with *η*_th_ of 30.54%, and *η*_ex_ of 35.43%, and *C*_tot_ reaches 6.278 × 10^8^ CNY, yielding an EPC of 0.61 CNY/kWh and a PDD of 32.65 years. As indicated by the PDD and EPC, the operation state of the PBC is extremely harsh under 30% load condition of gas turbine. However, *η*_th_ still remains at 30.54%, which further demonstrates the superiority of the PBC.

### 5.5. Performance Comparison of the PBC Under Variable Loads

Based on the above research, it is clear to find that the PBC shows different system performance under different gas turbine loads. This paper summarizes the optimal operating parameters and corresponding performance indicators of the PBC across the GTL range from 30% to 100%, based on the optimization results presented in Section 5.1, Section 5.2, Section 5.3 and Section 5.4, in Table 6. This comprehensive overview facilitates a clear comparison of the system’s behavior under variable-load conditions. In this section, the performance variation rules of the PBC under variable operation loads are compared and summarized.

**Figure 15:**
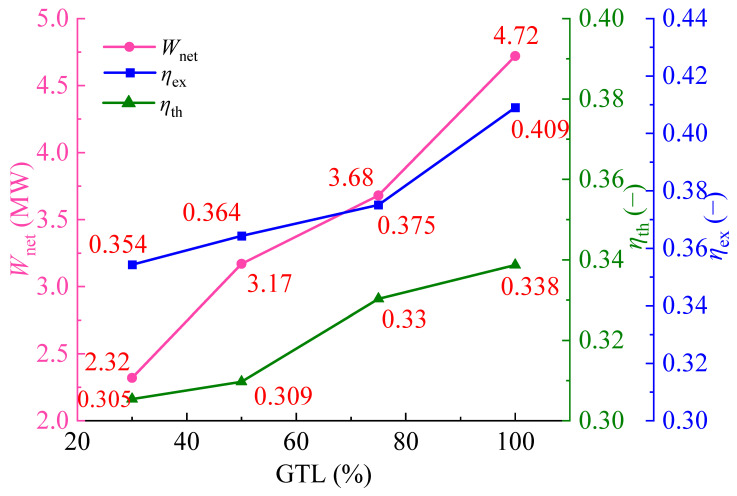
Variations of *W*_net_, *η*_th_, and *η*_ex_ of the PBC under variable GTLs.

Figure 15 shows the changes in *W*_net_, *η*_th_, and *η*_ex_ of the PBC as gas turbine load (GTL) varies from 30% to 100%. It is easy to see that *W*_net_, *η*_th_, and *η*_ex_ all show a gradually increasing trend with the increase in GTL. Among different parameters, *W*_net_ experiences the most pronounced variation, indicating that changes in GTL have the greatest influence on *W*_net_. With GTL ranging from 30% to 100%, *η*_ex_ and *η*_th_ of the PBC increase by 13.45% and 9.76%, respectively. Compared with 100% load, when GTL decreases from 75% to 30%, the related *W*_net_ decreases by 22%, 32.8%, and 50.8%, respectively. When the gas turbine operates at 30% load, *η*_th_ still reaches 30.5%, and *η*_ex_ reaches 35.4%. The superiority of the PBC is clearly shown even under low operation loads of the gas turbine.

**Figure 16:**
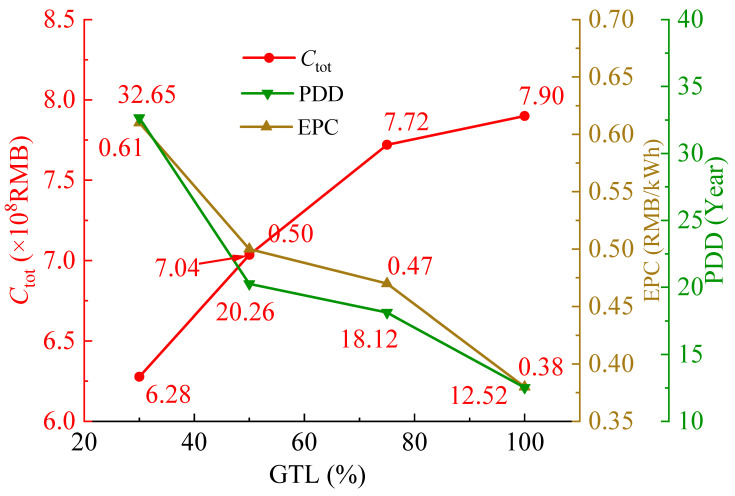
Variations of *C_tot_*, EPC, and PDD of the PBC under variable GTLs.

Figure 16 shows the variations in economic parameters of PBC as gas turbine load (GTL) varies from 30% to 100%. Generally, *C*_tot_ increases with the increase in GTL. This is because the increase in gas turbine load leads to an increase in *m*_CO2_, thereby causing an increase in *C*_tot_. With GTL increasing from 30% to 100%, *C*_tot_ increases by 20.63%. Due to more significant growth of *W*_net_, both the EPC and PDD of the PBC gradually decrease with GTL. When GTL increases from 30% to 100%, EPC and PDD decrease by 61.65% and 37.7%, respectively. When GTL = 30%, EPC exceeds 0.6 CNY/kWh, and PDD becomes extremely long, which implies that the economy of PBC is already significantly degraded. Therefore, it is recommended that the operation load proportion of the gas turbine is higher than 30%.

**Figure 17:**
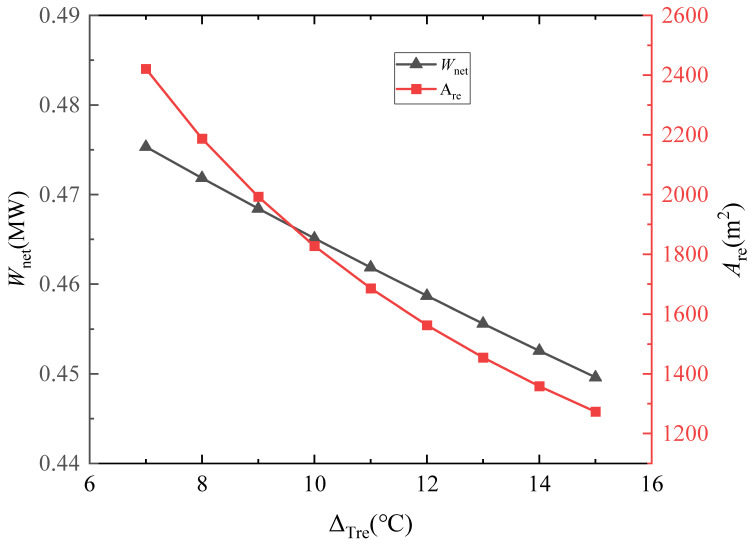
Effect of regenerator pinch temperature difference on *A*_re_ and *W*_net_.

As shown in Figure 17, with the decrease in the pinch temperature difference in the regenerator, *W*_net_ gradually increases, while the heat exchanger area rises sharply. Therefore, a smaller pinch temperature difference is not necessarily better, and factors such as power generation capacity, economic efficiency, and floor space must be comprehensively considered. Based on the above analysis and the same optimization logic, the pinch temperature difference in the regenerator is finally determined to be 10 °C, that of the preheater and heater is set to 20 °C, and that of the precooler is specified as 3 °C (to approach the critical temperature at the compressor inlet).

## 6. Conclusions

This study presents the PBC, a distinctive system configuration designed to overcome the performance limitations of an SBC in offshore platform gas turbine waste heat recovery. The originality of this work is demonstrated through the innovative parallel arrangement of the preheater, a comprehensive 3E analysis under variable loads, and the derivation of optimal operation strategies. The key findings are concluded as follows:

1. Adding a preheater to the SBC can effectively improve system performance and optimize economic indicators of the PBC. Compared with traditional SBCs, significant improvements are demonstrated in the thermodynamic performance of PBCs, with *W*_net_ and *η*_th_ increasing by 27.9% and 41.4% on average, and *η*_ex_ showing an average enhancement of 25.6%. Concurrently, economic metrics indicate an average 17.16% increase in *C*_tot_ and a 21.23% reduction in EPC, further highlighting the comprehensive superiority of PBCs over SBCs.
2. Parameters *W*_net_, *η*_th_, *C*_tot_, and *η*_ex_ of the PBC all increase with the rise in GTL, while both EPC and PDD decrease as GTL increases.
3. Compared to full load operation, when the load operation ratio of the gas turbine decreases from 75% to 30%, *W*_net_ decreases by approximately 22%, 32.8%, and 50.8%, respectively. When the GTL of the gas turbine increases from 30% to 100%, EPC and PDD decrease by 61.65% and 37.7%, respectively.
4. Under the harsh operating condition of 30% operation load, the EPC of the PBC under optimal working condition exceeded *C*_elec_. From an economic perspective, it is recommended that the load operation proportion of the gas turbine is higher than 30%. However, even under GTL = 30%, *η*_th_ and *η*_ex_ can still remain at 30.54% and 35.43%, indicating the feasibility of low load operation from a thermodynamic perspective.

This study confirms that the PBC significantly outperforms the SBC from a thermodynamic and economic perspective, indicating its good feasibility in the waste heat recovery sector. Meanwhile, the optimal operation parameters of PBC under variable operation loads of gas turbines are explored, and the performance variation rules of PBC during variable-load conditions are summarized. Future research will focus on the application scenario expansion and structural optimization of the SCO_2_ Brayton cycle, such as impact analysis on system performance after integrating a preheater or a recuperator. Future work will be developed along two main paths: At the cycle configuration level, a systematic comparison between PBCs integrated with advanced features and the simpler layout studied here will be conducted, alongside a comprehensive 3E benchmarking against mature gas turbine–Rankine combined cycles to clarify its techno-economic position. At the system integration level, research will focus on harnessing the waste heat from the PBC precooler, which holds a higher temperature potential than conventional cycles, evaluating its feasibility for cogeneration applications such as low-temperature process heat, desalination, or district heating, thereby advancing the concept from efficient power generation towards a comprehensive energy solution.

## Figures and Tables

**Table 1:** Settings of the main input parameters.

Parameter	Value	Reference
Turbine inlet pressure (TIP) (MPa)	12–20 ^b^	-
Compressor inlet pressure (CIP) (MPa)	8.2–9.0	-
Compressor inlet temperature (CIT) (°C)	36–41	[40]
Turbine inlet temperature (TIT) (°C)	297–377	[40]
Temperature difference at the recuperator and preheater nodes (Δ*T*i) (°C)	20 ^a^	-
Pressure drop on the CO_2_ side of the preheater and the heater (MPa)	0.2	[41]
Pressure drop on the flue gas side of the preheater and the heater (kPa)	3	[41]
Isentropic efficiency of the compressor	0.80	[42]
Isentropic efficiency of the turbine	0.83	[42]
Ambient temperature *T*_0_ (°C)	35	[39]
Cooling water pressure (kPa)	500	-

Note: ^a^: The pinch temperature difference of 20 °C is selected for the preheater and heater after comprehensive consideration of the operating scenario, economic efficiency, and power generation capacity. ^b^: The 12–20 MPa turbine inlet pressure range was selected as it represents the point of diminishing returns in net power gain while respecting the practical performance limits of currently available compressor technology for offshore applications.

**Table 2:** Balance equations for each system component.

Cycle	Component	Mass	Energy	Exergy
SBC	Compressor	${\dot{m}}_{1}={\dot{m}}_{2}$	${\dot{W}}_{\mathrm{com}}={\dot{m}}_{1}\times \left(h_{2s}-h_{1}\right)/\eta_{\mathrm{cs}}$	${\dot{E}}_{X_{D,\mathrm{com}}}={\dot{W}}_{\mathrm{com}}-\left({\dot{E}}_{x_{2}}-{\dot{E}}_{x_{1}}\right)$
	Recuperator	${\dot{m}}_{2}={\dot{m}}_{3}$	$Q_{\mathrm{re}}={\dot{m}}_{2}\times \left(h_{3}-h_{2}\right)$	${\dot{E}}_{X_{D,\mathrm{re}}}=\left({\dot{E}}_{x_{6}}-{\dot{E}}_{x_{5}}\right)-\left({\dot{E}}_{x_{3}}-{\dot{E}}_{x_{2}}\right)$
	Heater	${\dot{m}}_{3}={\dot{m}}_{4}$	$Q_{h}={\dot{m}}_{3}\times \left(h_{4}-h_{3}\right)$	${\dot{E}}_{X_{D,h}}=\left({\dot{E}}_{x_{7}}-{\dot{E}}_{x_{8}}\right)-\left({\dot{E}}_{x_{4}}-{\dot{E}}_{x_{3}}\right)$
	Turbine	${\dot{m}}_{4}={\dot{m}}_{5}$	${\dot{W}}_{t}={\dot{m}}_{4}\times \left(h_{4}-h_{5s}\right)\times \eta_{\mathrm{ts}}$	${\dot{E}}_{X_{D,t}}=\left({\dot{E}}_{x_{4}}-{\dot{E}}_{x_{5}}\right)-{\dot{W}}_{t}$
	precooler	${\dot{m}}_{6}={\dot{m}}_{1}$	$Q_{\mathrm{cool}}={\dot{m}}_{6}\times \left(h_{6}-h_{1}\right)$	${\dot{E}}_{X_{D,\mathrm{cool}}}=\left({\dot{E}}_{x_{6}}-{\dot{E}}_{x_{1}}\right)-\left({\dot{E}}_{x_{11}}-{\dot{E}}_{x_{10}}\right)$
PBC	Compressor	${\dot{m}}_{1}={\dot{m}}_{2}$	${\dot{W}}_{\mathrm{com}}={\dot{m}}_{1}\times \left(h_{2s}-h_{1}\right)/\eta_{\mathrm{cs}}$	${\dot{E}}_{X_{D,\mathrm{com}}}={\dot{W}}_{\mathrm{com}}-\left({\dot{E}}_{x_{2}}-{\dot{E}}_{x_{1}}\right)$
	Recuperator	${\dot{m}}_{\alpha }={\dot{m}}_{2}\times \alpha$	$Q_{\mathrm{re}}={\dot{m}}_{\alpha }\times \left(h_{3}-h_{2}\right)$	${\dot{E}}_{X_{D,\mathrm{re}}}=\left({\dot{E}}_{x_{6}}-{\dot{E}}_{x_{5}}\right)-\left({\dot{E}}_{x_{3}}-{\dot{E}}_{x_{2}}\right)$
	Preheater	${\dot{m}}_{\beta }={\dot{m}}_{2}-{\dot{m}}_{\alpha }$	$Q_{\mathrm{pre}}={\dot{m}}_{\beta }\times \left(h_{3}-h_{2}\right)$	${\dot{E}}_{X_{D,\mathrm{pre}}}=\left({\dot{E}}_{x_{8}}-{\dot{E}}_{x_{9}}\right)-\left({\dot{E}}_{x_{3}}-{\dot{E}}_{x_{2}}\right)$
	Heater	${\dot{m}}_{2}={\dot{m}}_{4}$	$Q_{h}={\dot{m}}_{4}\times \left(h_{4}-h_{3}\right)$	${\dot{E}}_{X_{D,h}}=\left({\dot{E}}_{x_{7}}-E_{x_{8}}\right)-\left({\dot{E}}_{x_{4}}-{\dot{E}}_{x_{3}}\right)$
	Turbine	${\dot{m}}_{4}={\dot{m}}_{5}$	${\dot{W}}_{t}={\dot{m}}_{4}\times \left(h_{4}-h_{5s}\right)\times \eta_{\mathrm{ts}}$	${\dot{E}}_{X_{D,t}}=\left({\dot{E}}_{x_{4}}-{\dot{E}}_{x_{5}}\right)-{\dot{W}}_{t}$
	Precooler	${\dot{m}}_{6}={\dot{m}}_{1}$	$Q_{\mathrm{cool}}={\dot{m}}_{6}\times \left(h_{6}-h_{1}\right)$	${\dot{E}}_{X_{D,\mathrm{cool}}}=\left({\dot{E}}_{x_{6}}-{\dot{E}}_{x_{1}}\right)-\left({\dot{E}}_{x_{11}}-{\dot{E}}_{x_{10}}\right)$

**Table 3:** Correction coefficients used in the component calculation [47].

Component	*K*_1_	*K*_2_	*K*_3_	*C*_1_	*C*_2_	*C*_3_	*B*_1_	*B*_2_	*F*_m_	*F*_bm_
Compressor	2.28	1.36	−0.10	/	/	/	/	/	1	1.5
Pump	3.38	0.05	0.15	−0.393	0.0396	−0.002	1.89	1.35	1.5	/
Turbine	2.24	1.49	−0.16	/	/	/	/	/	2.35	2.12
Heat exchanger	4.32	−0.30	0.16	0.038	−0.112	0.081	1.63	1.66	1.2	/

**Table 4:** AGT-12 gas turbine parameters at a temperature 35 °C and a pressure of 101.325 kPa.

Number	Parameter	Unit	Load Ratio			
			100%	75%	50%	30%
1	Genset power	kW	9009	6757	4505	2703
2	Exhaust temperature	°C	519	485	456	430
3	Exhaust flow rate	kg/s	53.16	50.5	45.8	42.0
4	Exhaust components (mol%)					
	O_2_	%	15.223	15.489	16.235	16.930
	CO_2_	%	2.917	2.778	2.388	2.025
	H_2_O	%	5.441	5.227	4.629	4.072
	N_2_	%	75.529	75.614	75.853	76.076
	Ar	%	0.891	0.892	0.895	0.897

**Table 5:** Input parameters in Chen’s [56] study.

Parameter	Value
CIT (°C)	45–65
CIP (MPa)	7.8
TIP (MPa)	25.03
TIT (°C)	500–700
Pressure ratio (−)	3.21
Compressor efficiency (%)	89
Turbine efficiency (%)	93
Pinch-point temperature difference (°C)	15
${\dot{m}}_{g}$ (kg/s)	67.1
${\dot{m}}_{\mathrm{water}}$ (kg/s)	262.8

**Table 6:** Summary of optimal operating parameters and performance indicators for the PBC under variable gas turbine loads.

Parameter	Unit	100% Load	75% Load	50% Load	30% Load
Optimal Operating Parameters					
Optimal TIP	MPa	18	16	18	16
Optimal CIP	MPa	8.6	8.8	8.4	8.6
Optimal CIT	K	310	310	309	309
Optimal TIT	K	600	600	580	580
Thermodynamic Performance					
*W*_net_	MW	4.72	3.68	3.17	2.32
*η*_th_	(−)	0.3387	0.3303	0.3097	0.3054
*η*_ex_	(−)	0.409	0.3751	0.3643	0.3543
Economic Performance					
*C*_tot_	10^8^ CNY	7.9	7.72	7.035	6.278
EPC	CNY/kWh	0.38	0.47	0.5	0.61
PDD	Years	12.52	18.12	20.26	32.65

## Data Availability

The original contributions presented in this study are included in the article. Further inquiries can be directed to the corresponding author.

## Abbreviations

The following abbreviations are used in this manuscript:

CEPCI	Chemical equipment plant cost index
CIP	Compressor inlet pressure, MPa
CIT	Compressor inlet temperature, °C
CRF	Capital recovery factor, (-)
EPC	Electricity production cost, CNY/kWh
GTL	Gas turbine load, %
MCT	Module Costing Technique
ORC	Organic Rankine Cycle
PBC	Parallel-preheating recuperated Brayton cycle
PDD	Dynamic payback period, Year
SBC	Simple recuperated Brayton cycle
TIP	Turbine inlet pressure, MPa
TIT	Turbine inlet temperature, °C
